# An emerging PB2-627 polymorphism increases the zoonotic risk of avian influenza virus by overcoming ANP32 host restriction in mammalian and avian hosts

**DOI:** 10.1128/jvi.00853-25

**Published:** 2025-08-27

**Authors:** Yuxin Guo, Sicheng Shu, Yong Zhou, Wenjing Peng, Zhimin Jiang, Yudong Li, Tian Li, Fanshu Du, Linlin Wang, Xue Chen, Jinze Dong, Chuankuo Zhao, Maggie Haitian Wang, Yipeng Sun, Honglei Sun, Lu Lu, Paul Digard, Kin-Chow Chang, Hui-Ling Yen, Jinhua Liu, Juan Pu

**Affiliations:** 1 National Key Laboratory of Veterinary Public Health Security, Key Laboratory for Prevention and Control of Avian Influenza and Other Major Poultry Diseases, Ministry of Agriculture and Rural Affairs, College of Veterinary Medicine, China Agricultural University 630101 , Beijing, China; 2 Centre for Clinical Research and Biostatistics, JC School of Public Health and Primary Care, The Chinese University of Hong Kong, Hong Kong SAR, China; 3 CUHK Shenzhen Research Institute, Shenzhen, China; 4 The Roslin Institute, University of Edinburgh 15551, Edinburgh, United Kingdom; 5 School of Veterinary Medicine and Science, University of Nottingham, Sutton Bonington Campus 6123, Loughborough, United Kingdom; 6 School of Public Health, Li Ka Shing Faculty of Medicine, The University of Hong Kong 25809, Hong Kong SAR, China; University of North Carolina at Chapel Hill, Chapel Hill, North Carolina, USA

**Keywords:** influenza virus, PB2 gene, host adaptation

## Abstract

Alterations in the PB2-627 domain of avian influenza virus (AIV) can potentially increase the risk of cross-host species infections in humans and mammals. Recently, there has been a rise in human cases of AIV infections without the presence of the known mammalian determinant PB2-E627K. Here, we identified a variant, PB2-627V, which has evolved in poultry and has contributed to the increase in human AIV infections. By screening global PB2 sequences, we discovered a new independent cluster of PB2-627V that emerged in the 2010s, prevalent in avian, mammalian, and human AIV isolates, including those of H9N2, H7N9, H3N8, 2.3.4.4b H5N1, and other subtypes. We functionally assessed its host adaptation, fitness, and transmissibility across three subtypes of AIVs (H9N2, H7N9, and H3N8) in different host models. PB2-627V combines the viral properties of avian-like PB2-627E and human-like PB2-627K, facilitating AIVs to efficiently infect and replicate in chickens and mice by utilizing both avian- and human-origin ANP32A proteins. Importantly, PB2-627V promotes efficient transmission between ferrets through respiratory droplets. Deep sequencing of passaged chicken and transmitted ferret viral samples indicates that PB2-627V remains stable across the two host species and shows a high potential for long-term prevalence in avian species. Thus, the PB2-627V mutation in AIVs can stably transmit through poultry and can overcome the cross-species barrier to infect humans. Given the global prominence of AIVs, it will be prudent to monitor influenza viruses for the PB2-627V mutation as a potential marker for zoonotic spread.

**IMPORTANCE:**

Avian influenza viruses (AIVs) are significant zoonotic pathogens. There is a rising trend of human cases of AIVs caused by a range of virus subtypes, including H9N2, H3N8, and H5N1 viruses. Thus, it is crucial to understand the underlying viral changes in AIVs that could result in zoonotic spread. We identify mutation PB2-627V as an emerging viral factor that confers dual ability to the virus to infect and adapt to mammalian and avian hosts, and virus transmissibility in ferrets. The presence of PB2-627V in multiple subtypes of AIVs has the potential to cause public health risk. We therefore propose that PB2-627V be included as a molecular marker to assess the zoonotic risk of AIVs.

## INTRODUCTION

Avian influenza viruses (AIVs) are critically important in the evolution of pandemic influenza viruses (1). Adaptive changes in AIVs allow breakthrough infections in mammalian species by altering viral interactions with host-specific factors (2–4). Current clade 2.3.4.4b highly pathogenic avian influenza H5Ny virus outbreaks have swept across continents, posing unprecedented panzootic threats to birds, mammals, and global public health (5). Other subtypes of AIVs have also been responsible for recurrent human infections (6). For example, H7N7 (7), H7N9 (8, 9), H10N8 (10), H5N6 (11), H10N3 (12), and H3N8 (13) viruses have caused over 1,600 documented human cases (11). Notably, most of these AIV subtypes possess internal (i.e., non-glycoprotein) genes that are derived from avian H9N2 viruses (8, 11–13). H9N2 virus is globally widespread in wild and domestic birds (14, 15) and has caused repeated human infections since 2015. In such interspecies transmission events, critical adaptive mutations often occur on the PB2 protein of AIVs (12, 16–19).

PB2 is one of three polymerase subunits of the influenza virus RNA-dependent RNA polymerase, crucial for genome transcription and replication (20). Specific amino acid changes have been identified in PB2 as key determinants of avian-to-mammalian adaptation, through its interaction with host acidic leucine-rich nuclear phosphoprotein 32 (ANP32) in avian and mammalian cells (21–24). Human ANP32 proteins lack a 33-amino-acid insertion found in avian ANP32, restricting the interaction between avian-like PB2 and human ANP32A (23). PB2-627K in human influenza viruses interacts with human ANP32A and ANP32B (huANP32A and huANP32B) to support viral polymerase function (22). On the other hand, PB2-627E in AIV can utilize avian ANP32A but not huANP32A or huANP32B (22). PB2-E627K amino acid substitution in AIVs promotes viral polymerase activity in mammalian cells by enhancing PB2 interaction with huANP32A and huANP32B (25). The PB2-627K genotype is frequently detected in AIVs isolated from human infection cases but rarely detected in AIVs isolated from poultry or wild birds (26). In this study, we detected an emerging PB2-627 polymorphism, PB2-627V, in diverse subtypes, which can potentially increase zoonotic risk of AIVs by overcoming ANP32 host restriction in mammalian hosts.

## RESULTS

### PB2-627V prevalence in influenza virus isolates from avian and mammalian species

**Fig 1:**
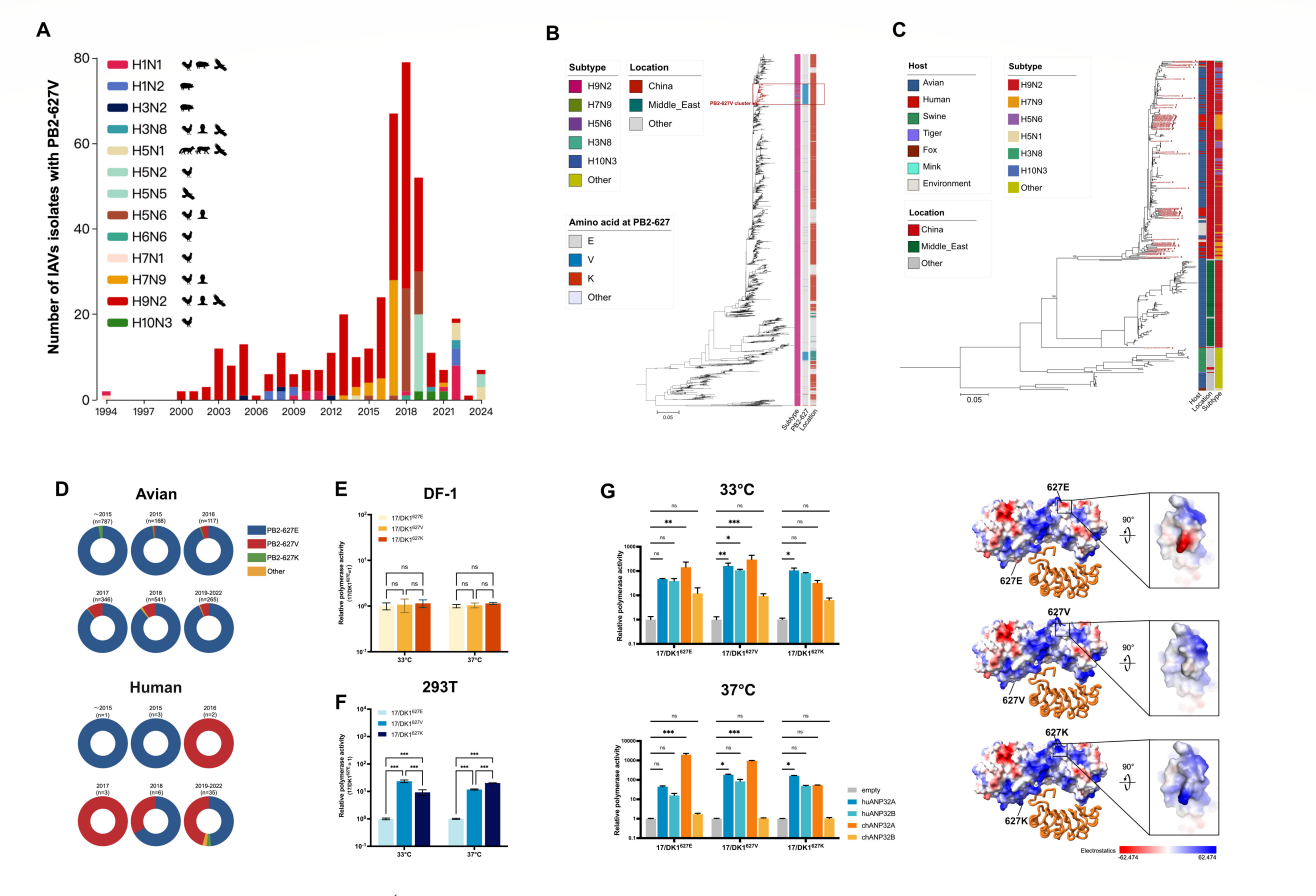
Emergence and cross-host species function of PB2-627V. (**A**) The global prevalence of PB2-627V in influenza A viruses (IAVs) (excluding human H3N2 and H1N1pdm09 viruses) during 1994–2024. Bars show the number of isolates by subtype and host. (**B**) Phylogenetic tree of PB2 genes of H9N2 with different amino acid residues at PB2-627 (*n* = 4,523). (**C**) Phylogenetic tree of PB2 genes with the residue 627V collected worldwide during 1994–2022 (*n* = 394). Human isolates’ names are in red. Other included H1N1, H1N2, H5N2, H5N2, and H6N6. (**D**) Amino acid polymorphisms of the PB2-627 residue in avian and human-derived H9N2 viruses isolated in China. (**E and F**) Relative polymerase activities at 33°C or 37°C. DF-1 cells (**E**) and 293T cells (**F**) were transfected with firefly minigenome reporter, *Renilla* expression control, and polymerase (PB2, PB1, PA, and NP) of 17/H9N2^627E^, 17/H9N2^627V^, or 17/H9N2^627K^. Cells were incubated for 24 hours, and the luciferase activities, normalized to corresponding *Renilla* luciferase activity, are presented relative to 17/H9N2^627E^ (set as 1). Each data point is the mean ± SD of three independent experiments (one-way analysis of variance). (**G**) Relative polymerase activities in DKO cells overexpressing ANP32 proteins. chANP32A, chANP32B, huANP32A, huANP32B, or empty vector was transfected into DKO cells, incubated at 33°C and 37°C. After 24 hours, cells were transfected with firefly minigenome reporter, *Renilla* expression control, and polymerase genes of 17/H9N2^627E^, 17/H9N2^627V^, or 17/H9N2^627K^. Luciferase activity was assayed, normalized to corresponding *Renilla* luciferase activity, and presented relative to empty vector (set as 1). Each data point is the mean ± SD of three independent experiments. Statistical significance was based on two-way analysis of variance and subsequent Dunnett’s test, compared with the corresponding value of empty vector (ns, not significant; *P < 0.05; **P < 0.01; and ***P < 0.001). (**H**) Impact of amino acids at the PB2-627 position on the surface charge of the PB2 protein, huANP32A, is displayed as a cartoon. Red (negative) and blue (positive) residues are shown.

Given the crucial role of PB2 residue 627 in host adaptation, we analyzed 42,297 influenza PB2 sequences reported worldwide (excluding human H3N2 and H1N1pdm09 viruses) for polymorphisms at this position. We found that the number of sequences with PB2-627V increased rapidly after 2016 (Fig. 1A; Fig. S1). Among the viruses with PB2-627V, 71.3% were isolated from avian species, 14.7% were from humans, and 14.0% were from other mammalian hosts (such as fox, swine, tiger, and mink) and the environment (Fig. 1A; Table S1). In geographical distribution, 61.7% of the viruses were isolated in China, with the remaining (38.3%) found in the Middle East, Germany, Mexico, Canada, Italy, Spain, Russia, America, Denmark, and Belgium (Table S1). In terms of subtype distribution, 64.5% of PB2-627V variants were detected in H9N2 viruses, 9.9% in H7N9 viruses, and 9.1% in H5N6 viruses; the remaining 16.5% were in different subtypes: H1N1, H1N2, H3N2, H3N8, H5N1, H5N2, H6N6, H7N1, and H10N3 (Fig. 1A; Table S1). Notably, PB2-627V has also been identified in clade 2.3.4.4b H5N1 viruses isolated from wild birds and red foxes (Fig. 1A; Table S1).

Phylogenetic analysis of the H9N2-subtype PB2 gene showed an independent cluster emerged through the acquisition of the PB2-E627V mutation, which we herein designate as the PB2-627V cluster (Fig. 1B). Subsequent analysis of all PB2 genes with PB2-627V from different subtypes showed that most human and avian isolates from H9N2 and its reassortants (H3N8, H5N1, H5N6, H7N9, and H10N3 viruses) were grouped together (Fig. 1C). Time-scaled ancestral host reconstruction further indicated that the chicken-derived variant served as the ancestral source for human isolates (Fig. S2). Notably, among human isolates of H9N2 virus, 627V occurred at a frequency greater than in avian isolates during 2016–2022 (Fig. 1D). Similarly, there was a dramatic increase in PB2-627V detection in avian and human hosts for the H7N9, H5N6, and H3N8 subtypes after 2016 (Fig. S3). Thus, PB2-627V appears as an emerging adaptation of AIVs in mammalian and avian hosts.

### PB2-627V promotes polymerase activity of H9N2 AIV in human and chicken cells by utilizing ANP32 proteins of both host species

To evaluate the functionality of PB2-627V, we used a previously identified chicken H9N2 virus A/chicken/Hubei/HB17/2017 (referred to as 17/H9N2^627E^) with PB2-627E as the backbone and introduced the PB2-E627V and PB2-E627K changes. Although PB2-627V and -627K are found in chicken isolates, this approach allowed the dissection of the function of this single mutation. A dual-luciferase assay system was used to compare viral polymerase activities conferred by PB2-627E, V, and K in avian DF-1 cells and human 293T cells at 33°C and 37°C, respectively, mimicking the temperature of the human upper and lower respiratory tract (27). In DF-1 cells, 17/H9N2^627E^, 17/H9N2^627V^, and 17/H9N2^627K^ showed comparable polymerase levels at 33°C and 37°C (Fig. 1E; Fig. S4). However, a 23.6-fold (±2.6) increase in polymerase activity at 33°C and an 11.8-fold (±0.6) increase at 37°C were found with 17/H9N2^627V^ in 293T cells compared with 17/H9N2^627E^ (*P* < 0.001); 17/H9N2^627K^ exhibited a 9.3-fold (±2.6) increase in polymerase activity at 33°C and a 20.2-fold (±0.4) increase at 37°C (*P* < 0.001) relative to 17/H9N2^627E^ (Fig. 1F; Fig. S4). In avian DF1 cells, PB2-627V conferred more robust viral transcription and replication than PB2-627K, while in human A549 cells, it surpassed those of PB2-627E (Fig. S5A and B).

Viral polymerase activity was further examined in ANP32A and ANP32B double-knockout 293T (DKO) cells over-expressing chicken- or human-ANP32 protein(s) (25). As expected, polymerase activities of all three viruses (17/H9N2^627E^, 17/H9N2^627V^, and 17/H9N2^627K^) in control DKO cells were significantly lower than those in the wild-type 293T cells (Fig. S5C). Polymerase activity of 17/H9N2^627E^ in the DKO cells was significantly enhanced in the presence of chicken ANP32A (chANP32A) at both 33°C and 37°C (*P* < 0.01) (Fig. 1G; Fig. S6A). With 17/H9N2^627V^, huANP32A and chANP32A in DKO cells both significantly enhanced the polymerase activity at 33°C and 37°C (*P* < 0.05), and huANP32B significantly enhanced polymerase activity only at 33°C (*P* < 0.05) (Fig. 1G; Fig. S6A and B). With 17/H9N2^627K^, the expression of huANP32A in DKO cells significantly enhanced viral polymerase activity at 33°C and 37°C (*P* < 0.05) (Fig. 1G; Fig. S6A and B).

ANP32 facilitates the assembly and stabilization of the ternary replicase-ANP32-encapsidase replication complex by acting as an electrostatic chaperone-disaggregase for apo-FluPol (22, 28). This activity is mechanistically linked to a distinct positively charged pathway at the PB2-NLS (encapsidase)/PB2-627 (replicase) interface, which selectively binds the negatively charged proximal LCAR region of huANP32A/B. In contrast, avian FluPol exhibits a mixed surface that preferentially binds the avian ANP32A LCAR—a structural adaptation enabled by a 33-residue insertion (29, 30). Based on the published structure of the asymmetric polymerase dimer-ANP32A complex (PDB:8RMR), we introduced mutations and calculated their electrostatic status. Our analysis revealed that the PB2-E627V mutation, although failing to establish a fully continuous positively charged pathway like PB2-E627K, partially reversed the negative charge surface caused by the wild-type PB2-627E residue (Fig. 1H). In summary, these findings indicate that PB2-627V can utilize chicken and human ANP32A in the respective species of cells to facilitate viral polymerase activity.

### PB2-627V in the H9N2 virus maintains efficient replication in chickens and facilitates replication in human cells and mice

**Fig 2:**
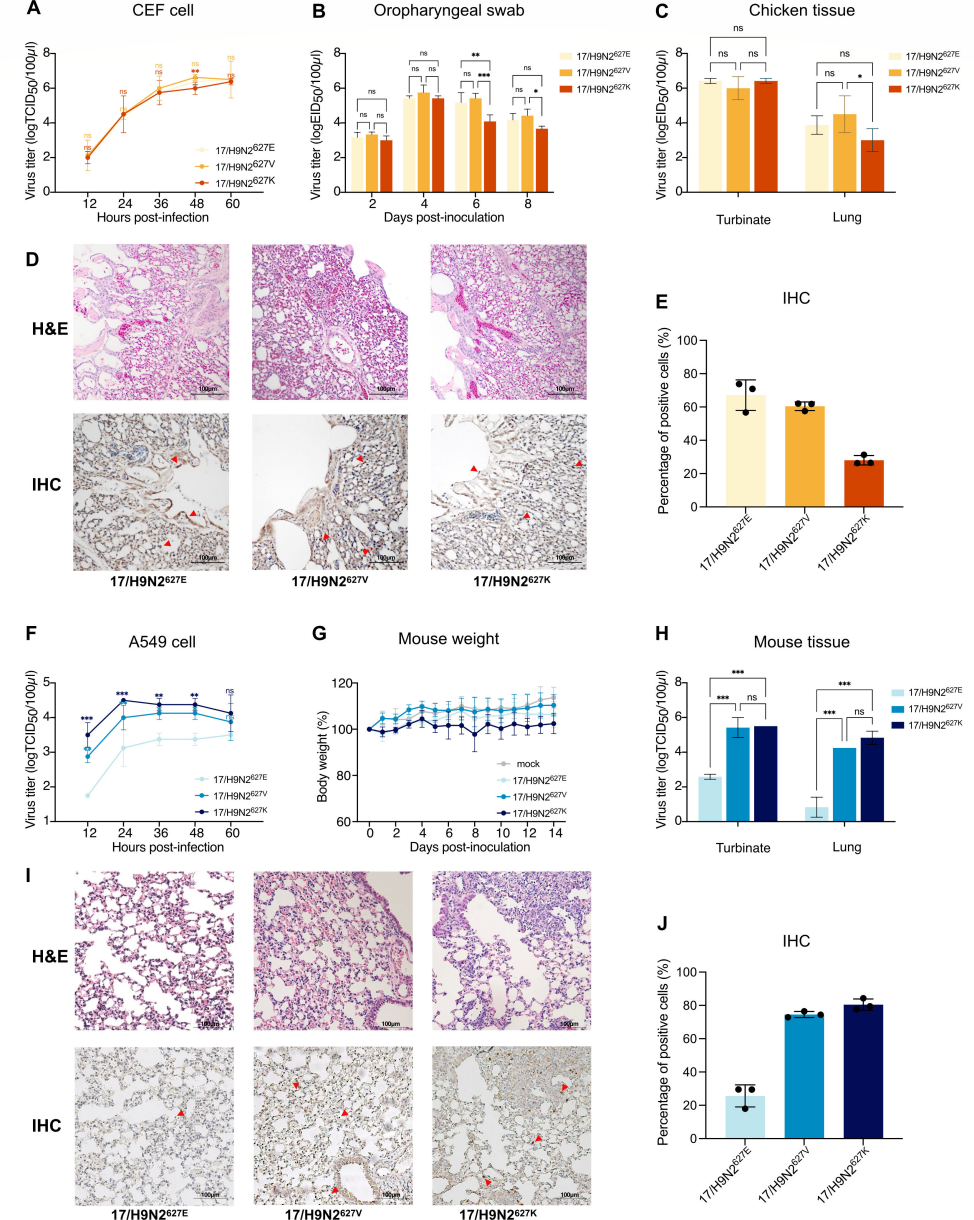
PB2-627V conferred dual adaptability to the H9N2 virus to replicate in avian and mammalian species. (**A**) CEF cells were infected with the indicated viruses at 0.01 multiplicity of infection, and virus titers were determined at the indicated times post-infection by TCID_50_ assays. (**B–E**) Groups of six chickens were intranasally infected with indicated viruses at 10^6^ EID_50_, three chickens from each group were used to determine the viral titers (**B**) from oropharyngeal samplings at 2, 4, 6, and 8 dpi. Another three chickens from each group were euthanized at 3 dpi. Virus titers (**C**) of the turbinates and lungs were determined. Representative histopathological findings (**D**) were determined in the lungs of infected chickens. Lung sections were stained with hematoxylin and eosin (H&E) (upper) and by immunohistochemistry (IHC) against influenza viral NP antigen (lower). Scale bars, 100 mm. (**E**) The percentage of IHC-positive cells. (**F**) A549 cells were infected with the indicated viruses at 0.1 multiplicity of infection, and virus titers were determined by TCID_50_ assays at indicated times post-infection. (**G–J**) Groups of eight mice were infected with the indicated virus at doses of 10^6^ TCID_50_. Five mice from each group were monitored for body weight changes (**G**) over 14 days. An additional three mice from each group at 3 dpi were euthanized. Virus titers (**H**) in the turbinates and lungs were determined by TCID_50_ assays. Representative histopathological findings (**I**) were determined in the lungs of infected mice. Lung sections were stained with H&E (upper) and by IHC against influenza viral NP antigen (lower). Scale bars, 100 mm. (**J**) The percentage of immunohistochemistry-positive cells. Data are represented as mean ± SD. Statistical significance was based on two-way analysis of variance, compared with the corresponding value of 17/H9N2^627E^ (ns, not significant; *P < 0.05; **P < 0.01; and ***P < 0.001).

Next, viruses with the three PB2 627 polymorphisms were rescued on the background of the 17/H9N2 virus. The replication kinetics of these 17/H9N2^627E^, 17/H9N2^627V^, and 17/H9N2^627K^ viruses were assessed in avian CEF cells. Virus titers of 17/H9N2^627K^ were significantly lower than that of 17/H9N2^627E^ at 48 hours post-infection (hpi) (*P* < 0.01), and no significant difference was detected between 17/H9N2^627V^ and 17/H9N2^627K^ (*P* > 0.05, Fig. 2A). Chickens individually inoculated with 10^6^ EID_50_ of each virus showed no mortality (Fig. S7A) and no apparent clinical signs. Viral loads detected from oropharyngeal swabs of 17/H9N2^627V^ and 17/H9N2^627E^ inoculated chickens were significantly higher than those infected with 17/H9N2^627K^ at 6 and/or 8 days post-infection (dpi) (*P* < 0.05, Fig. 2B). In chickens, the three viruses replicated to comparable titers in the turbinates at 3 dpi, but 17/H9N2^627V^ replicated to higher titers than 17/H9N2^627K^ in the lungs (*P* < 0.05, Fig. 2C). Histopathological examination of chicken lungs at 3 dpi indicated that all viruses induced inflammatory responses, with more noticeable hemorrhage in 17/H9N2^627V^-infected lungs (Fig. 2D); 17/H9N2^627E^ and 17/H9N2^627V^ viruses produced more viral NP protein-positive cells in the lung than in corresponding 17/H9N2^627K^ infected chickens (Fig. 2D and E). These findings indicate that PB2-627V and PB2-627E replicated comparably well in chickens.

In infected human A549 cells, virus titers of 17/H9N2^627V^ and 17/H9N2^627K^ were significantly higher than those of 17/H9N2^627E^ from 12 to 48 hpi (*P* < 0.001); there was no significant difference between 17/H9N2^627V^ and 17/H9N2^627K^ (*P* > 0.05, Fig. 2F). Mice inoculated with 17/H9N2^627E^, 17/H9N2^627V^, or 17/H9N2^627K^ virus showed no obvious clinical signs, as in infected chickens (Fig. 2G; Fig. S7B). Higher virus loads were detected in the nasal turbinates and lungs of mice infected with 17/H9N2^627V^ and 17/H9N2^627K^ at 3 dpi than those with 17/H9N2^627E^ (*P* < 0.001); there was no significant difference in virus titers between the 17/H9N2^627V^ and 17/H9N2^627K^ groups (*P* > 0.05, Fig. 2H). Likewise, 17/H9N2^627V^ and 17/H9N2^627K^ viruses produced more viral NP protein-positive cells in the lung than in corresponding 17/H9N2^627E^-infected mice (Fig. 2I and J). Thus, the infectivity of the PB2-627V H9N2 virus in human cells and mice is similar to that of the PB2-627K virus.

### PB2-627V confers aerosol transmission of H9N2 virus in ferrets

**Fig 3:**
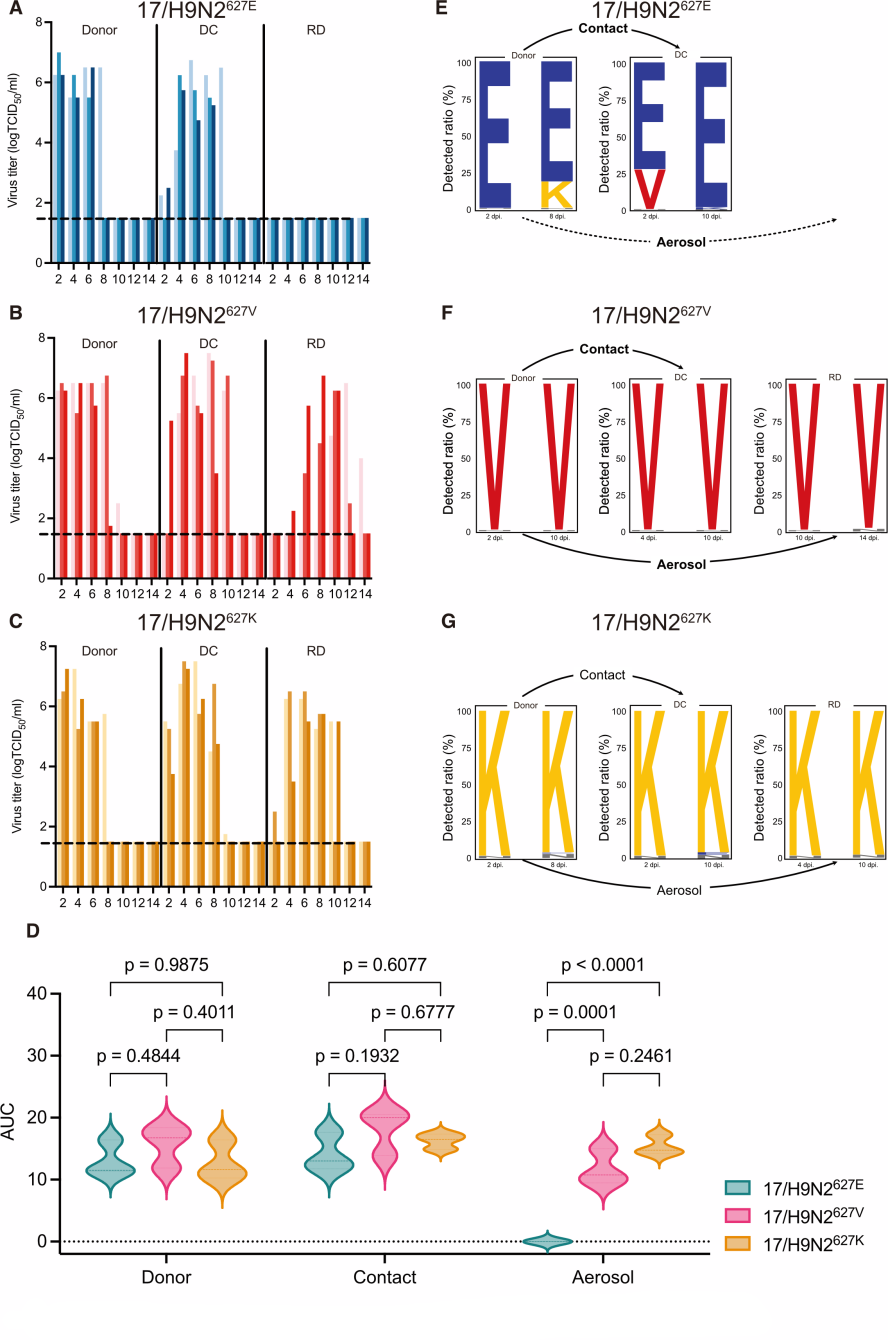
PB2-627V facilitated aerosol transmissibility of H9N2 virus in ferrets. Each ferret (*n* = 3) was inoculated with 10^6^ TCID_50_ of the indicated virus. The next day, the infected ferret was individually paired by co-housing with a DC ferret; an RD-contact ferret was also housed in a wire frame cage adjacent to the infected ferret. Nasal washes were collected for virus-shedding detection every other day from all ferrets from 2 dpi. (**A–C**) Virus titers of nasal washes were determined by TCID_50_ assays on MDCK cells. Each color bar represents virus titers of an individual ferret. Dashed lines indicate the lower limit of virus detection (10^1.5^ TCID_50_/mL). (**D**) AUC values approximate total virus output throughout infection (mean AUC from three ferrets). (**E–G**) For infected or transmitted ferrets, the nasal washes collected at the first and last virus detection time were analyzed by deep sequencing. Detection time points are based on the results shown in Table S4. The height of each letter indicates the relative ratio of amino acids at PB2-627. Sequencing was performed on an Illumina NovaSeq platform.

The transmissibility of influenza viruses between ferrets is an indicator of potential epidemic or even pandemic risk. Direct contact (DC) and respiratory droplet (RD) transmission tests of the 17/H9N2^627E^, 17/H9N2^627V^, and 17/H9N2^627K^ viruses were performed in ferrets. All viruses infected the directly inoculated donor animals, as assessed by virus titers in nasal washes (Fig. 3A through C; Table S2). The 17/H9N2^627V^ and 17/H9N2^627K^ viruses were transmitted to all DC ferrets and all RD ferrets, but 17/H9N2^627E^ was only transmitted to DC groups. Transmission by RD in the 17/H9N2^627V^ group took place around 2 days later than in the 17/H9N2^627K^ group (Fig. 3A through C). Areas under the curve (AUC), based on virus titers over time in the nasal washes, were determined to compare total viral shedding during infection (Fig. 3D; Table S3). In donor ferrets, virus production was comparable between the three viruses. In DC ferrets, 17/H9N2^627V^ virus output (AUC = 18.13 ± 3.69) was slightly higher than that of 17/H9N2^627E^ virus (14.13 ± 3.01, *P* = 0.19) and 17/H9N2^627K^ virus (16.25 ± 1.27, *P* = 0.68), but there was no significant difference between the three groups. In RD ferrets, there was no significant difference in AUC between the 17/H9N2^627V^ group (AUC = 11.75 ± 2.88) and the 17/H9N2^627K^ group (15.42 ± 1.60, *P* = 0.25) (Fig. 3D; Table S3). Thus, like PB2-E627K, PB2-E627V promotes the aerosol transmissibility of the H9N2 virus in ferrets.

To monitor any polymorphic changes at residue PB2-627 during virus infection and transmission, next-generation sequencing was performed on nasal wash samples from #1 ferret of three groups (Table S3) at the first and the last time points of positive virus detection. The donor ferret infected with 17/H9N2^627E^ was first detected positive for the virus at 2 dpi (Fig. 3A), and the sequencing confirmed the E residue was dominant at the PB2-627 site (Fig. 3E). Following viral adaptation in the ferrets, sequencing of the donor ferret at 8 dpi revealed the emergence of the PB2-E627K mutation (17.18%). For the contact ferret, low viral titers were detected at 2 dpi (24 hours after contact with the donor ferret), with sequencing results indicating contact transmission enriched a higher proportion of PB2-E627V mutations (27.79%). However, by 10 dpi, the virus in the nasal swabs of the contact ferrets remained predominantly PB2-627E (Fig. 3E). In contrast, the PB2-627 residue of 17/H9N2^627V^ and 17/H9N2^627K^ viruses showed no significant mutation in the donor, DC, and RD ferrets (Fig. 3F and G). These results suggest that residue PB2-627V stably confers aerosol transmission of the H9N2 virus in ferrets.

### PB2-627V has potential for sustained prevalence in poultry

**Fig 4:**
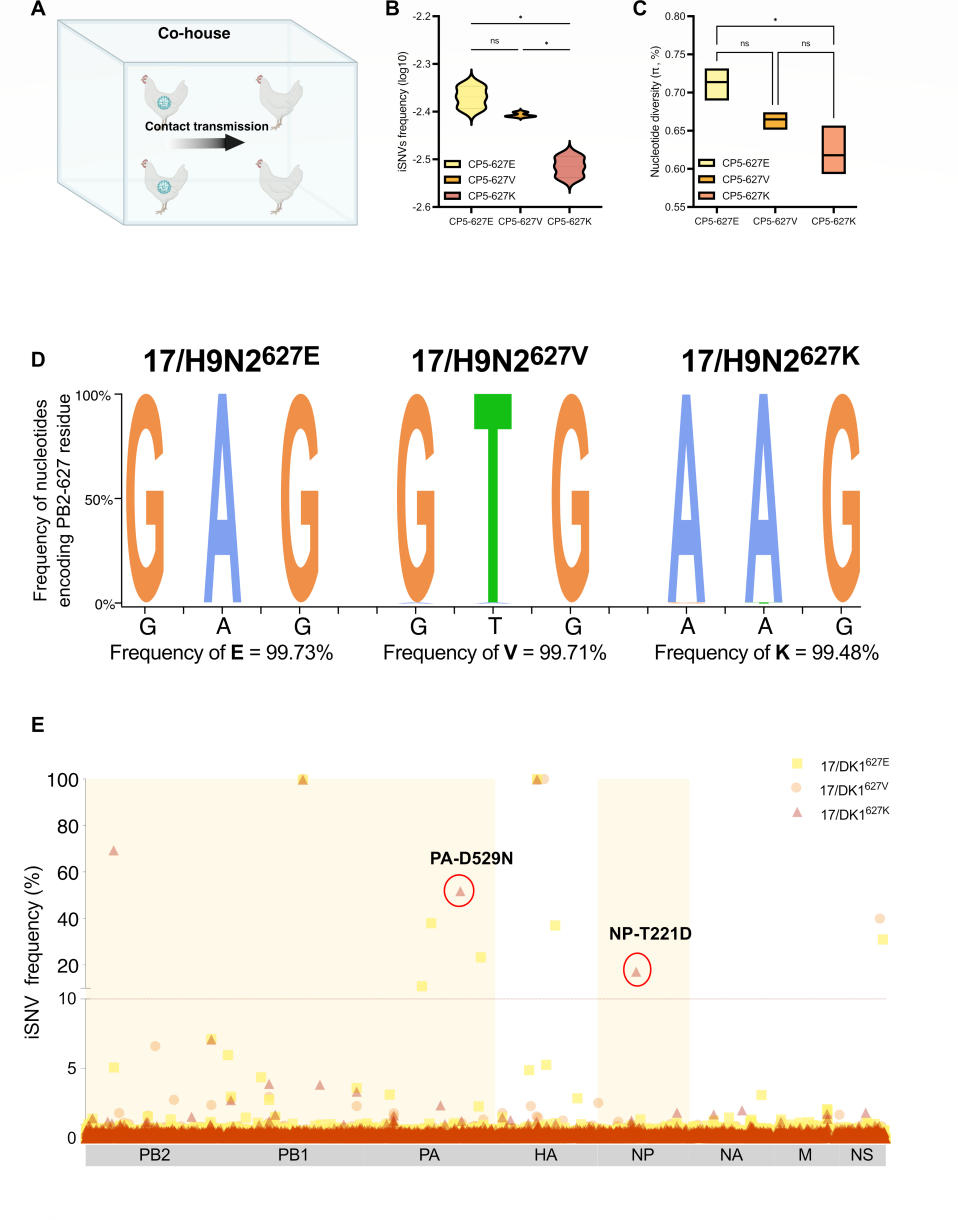
PB2-627V has the potential for sustained prevalence in poultry. (**A**) Schematic depiction of virus passage in chickens. Groups of two chickens were each intranasally infected with 10^6^ TCID_50_ of the indicated virus (**P1**) and subsequently passaged (**P1–P5**) by contact. (**B**) Intra-host single nucleotide variant frequency of P5 chickens. Variants of nucleotide sites detected above 1% threshold were identified as iSNVs. iSNV frequency of the whole genome was calculated by using the number of iSNVs divided by the total number of genome sites. iSNV frequency was expressed as the log. (**C**) The nucleotide diversity (π) of P5 chickens. (**D**) Variant frequency of the nucleotides encoding PB2-627 residue. The height of each letter indicates the average ratio of nucleotides encoding PB2-627 residue in two samples of each group. (**E**) Graphs represent all iSNVs detected in P5 chicken. Variant frequencies are plotted by genome location and are colored by virus groups. Two high-frequency non-synonymous iSNVs are circled and labeled with the corresponding amino acid mutations. Yellow backgrounds depict RNP genes (PB2, PB1, PA, and NP). Sequencing was performed on an Illumina NovaSeq platform. Data are presented as mean ± SD. Statistical significance was based on two-way analysis of variance (ns, not significant; *P < 0.05; **P < 0.01; and ***P < 0.001).

The 17/H9N2^627E^, 17/H9N2^627V^, and 17/H9N2^627K^ H9N2 viruses were serially passaged in chickens to assess the potential for sustained prevalence of PB2-627V in poultry. Chickens were directly infected with each virus (Passage 1, P1) and subsequently (P2–P5) passaged through contact transmission (Fig. 4A). All viruses were successfully passaged from P1 to P5. The proportion of intra-host single nucleotide variants (iSNVs) across the genome reflects the relative abundance of nucleotide variations under host-induced selective pressures, indicating viral genetic diversity within the host (31, 32). We performed next-generation sequencing and analyzed the iSNV of whole viral genomes from the lung samples of P5 chickens. The results revealed that 17/H9N2^627E^ and 17/H9N2^627V^ viruses exhibited significantly higher iSNV frequencies than 17/H9N2^627K^ (*P* < 0.05; Fig. 4B). Comparison of nucleotide diversity (π) after serial passaging demonstrated that the chicken host bottleneck had no significant impact on nucleotide diversity in 17/H9N2^627V^ (*P* > 0.05, Fig. 4C), but significantly reduced diversity in 17/H9N2^627K^ (*P* < 0.05, Fig. 4C).

At the amino acid level, the variant frequency at residue PB2-627 was below the 1% threshold in all P5 chicken samples (Fig. 4D). Although several high-frequency iSNVs (>10%) were detected in three groups, the vast majority were synonymous mutations. Only two non-synonymous variant residues (PA-D529N and NP-T211D) occurred at a high frequency in the 17/H9N2^627K^ group (Fig. 4E), suggesting that, compared with PB2-627E and PB2-627V, PB2-627K required other cooperative mutations in RNP genes for transmission in chickens. Taken together, PB2-627V showed a high potential for sustained prevalence in chickens.

### PB2-627V confers dual ability to replicate in chickens and mice to avian H7N9 and H3N8 viruses

We determined if the dual host adaptability of PB2-627V, detected at high frequency in the H9N2 virus, is transferable to other AIV subtypes. H7N9 and H3N8 viruses possess the PB2 gene and other internal genes derived from the H9N2 viruses (9, 33). The natural mutations PB2-E627V and PB2-E627K were separately introduced into the LPAI H7N9 (A/chicken/China/0606-12/2017, referred to as 17/H7N9^627E^) and H3N8 (A/chicken/Anhui/FE12/2022, referred to as 22/H3N8^627E^) (34) viruses. The resulting PB2-627V, PB2-627K, and PB2-627E viruses were assessed for replication in chickens and mice.

**Fig 5:**
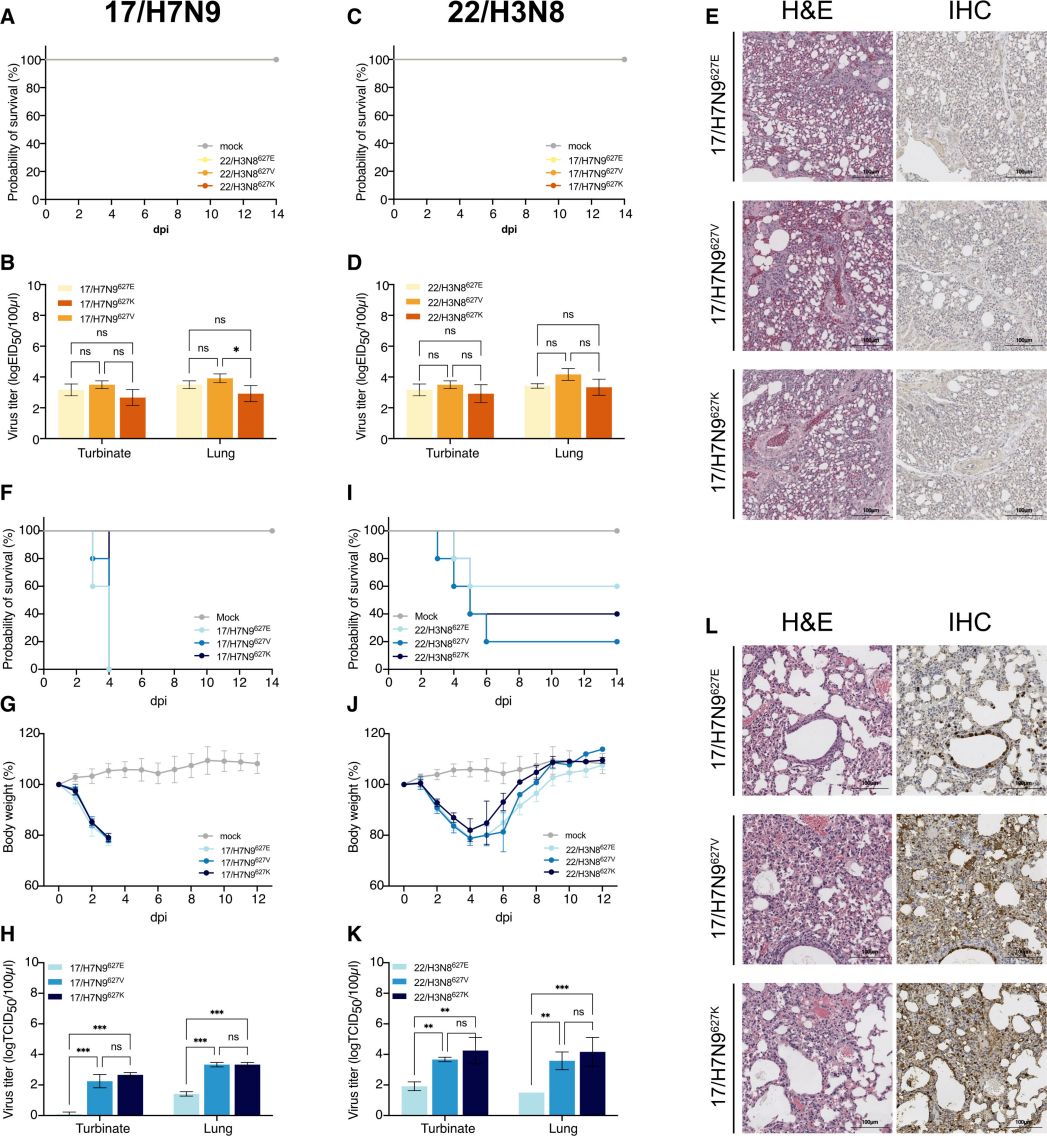
PB2-627V conferred dual adaptability in avian H7N9 and H3N8 viruses to replicate in chickens and mice. (**A–E**) Groups of six chickens were infected with indicated viruses at doses of 10^6^ EID_50_. Three infected chickens in each group were monitored for survival (**A and C**) over 14 days. At 3 dpi, the other three chickens in each group were euthanized. Virus titers (**B and D**) in the turbinates and lungs were determined by TCID_50_ assays. Representative histopathological findings (**E**) were determined in the lungs of infected chickens. Lung sections were stained with hematoxylin and eosin (H&E) (left) and by immunohistochemistry against influenza viral NP antigen (right). Scale bars, 100 mm. (**F–L**) Groups of eight mice were infected with indicated viruses at doses of 10^6^ TCID_50_. Five mice in each group were monitored for survival (**F and I**) and body weight changes (**G and J**) over 14 days. Mortality from infection included mice euthanized at a weight loss of 25% or more. At 3 dpi, the other three mice in each group were euthanized. Virus titers (**H and K**) in the turbinates and lungs were determined by TCID_50_ assays. Representative histopathological findings (**L**) were determined in the lungs of infected mice. Lung sections were stained with H&E (left) and by immunohistochemistry against influenza viral NP antigen (right). Scale bars, 100 mm. Data presented are represented as mean ± SD. Statistical significance was based on two-way analysis of variance (ns, not significant; *P < 0.05; **P < 0.01; and ***P < 0.001).

In chickens, introduction of PB2-E627V or PB2-E627K mutation into 17/H7N9 and 22/H3N8 viruses did not significantly affect replication or pathogenicity relative to their PB2-627E counterparts (Fig. 5A through E), although the replication of 17/H7N9^627V^ in chicken lungs was significantly higher than that of 17/H7N9^627K^ (*P* < 0.05) (Fig. 5B). Thus, PB2-E627V had little effect on virus replication and pathogenicity in chickens (Fig. 5A through E; Fig. S8A). In mice, no significant difference in weight loss or mortality was observed among the three groups, which may be attributed to the high-dose challenge. However, PB2-E627V and PB2-E627K mutations in 17/H7N9 and 22/H3N8 viruses increased the viral loads in the nasal turbinates and lungs (*P* < 0.01) (Fig. 5F through K). Murine lungs at 3 dpi with subtypes housing PB2-627V showed more severe pathology than those with PB2-627E (Fig. 5L for 17/H7N9; Fig. S8B for 22/H3N8). In summary, PB2-E627V significantly conferred increased lung pathology and replication of H7N9 and H3N8 virus subtypes in mice and maintained their replication fitness in chickens.

## DISCUSSION

Here, we identified PB2-627V, an emerging variant in AIVs, as a potential zoonotic risk factor. We observed that PB2-627V enhances the polymerase activity of H9N2 AIV in both human and chicken cells by utilizing ANP32A proteins from both species, thereby overcoming the restriction imposed by human ANP32 on AIVs carrying PB2-627E and exhibiting properties similar to those with PB2-627K. PB2-627V in H9N2 virus maintains efficient replication in chickens, facilitates virus replication in mice, and confers aerosol transmission in ferrets. Furthermore, PB2-627V is stably maintained in passages through chickens and also conferred the dual ability to replicate in chickens and mice to avian H7N9 and H3N8 viruses. Thus, the increased prevalence of this dual adaptive mutation of PB2-E627V in poultry could increase the likelihood of AIV infections in humans. In addition, the wide distribution of this mutation in multiple subtypes, hosts, and countries could pose global public health risks.

Phylogenetic analysis revealed that residue PB2-627V originated in chicken influenza viruses (likely the H9N2 subtype) and was subsequently transmitted to humans. The prevalence of PB2-627V in AIVs is on the rise since 2015, particularly within the H9N2 subtype; PB2-627V is also found in other subtypes such as H7N9, H5N6, H3N8, and H10N3, which harbor reassorted internal genes derived from H9N2 virus (8, 11–13). Serial passage studies indicate that compared with 17/H9N2^627E^ and 17/H9N2^627V^, 17/H9N2^627K^ exhibits greater nucleotide diversity in chicken hosts. Furthermore, 17/H9N2^627K^ acquired additional mutations in the polymerase complex during adaptation in chickens. Using the H7N9 virus with a V/K/E polymorphism at position 627, Luk et al. (35) observed that E627 and V627 were transmitted among chickens, but PB2-627K transmission is limited. These findings likely reflect residual functional constraints imposed by the PB2-627K mutation on viral transmission within avian hosts and explain why PB2-627K is very rare in avian isolates (36). Conversely, the PB2-627V cluster originated from avian-derived H9N2 PB2 genes and exhibits a high propensity for sustained circulation in poultry populations. Such persistent circulation heightens concerns about viral zoonotic spillover to human populations.

Cellular ANP32 proteins specifically support influenza virus genome replication. ANP32A can bridge PB2 dimers and form extensive contacts with two polymerases to maintain a dimeric assembly (22, 37). The C-terminal low-complexity acidic region (ANP32A^LCAR^) inserts between two PB2-627 domains of the polymerase dimer. Human ANP32A^LCAR^ contains only acidic amino acid residues. Thus, with human ANP32A, basic or uncharged residues (such as lysine, K, or valine, V) are preferred in the PB2-627 domain over acidic amino acids (such as glutamic acid, E) (21, 22). In avian cells, the additional 33 amino acids in chANP32A^LCAR^ allow AIVs carrying PB2-627E to effectively utilize chANP32A in avian cells (23, 38). Indeed, in the context of the H9N2 virus, we found that the polymerase activity of PB2-627V in avian cells is comparable to PB2-627E, and in human cells, it is comparable to PB2-627K, which suggests that the nonpolar neutral amino acid of PB2-627V has the dual ability to interact with avian ANP32A and human ANP32A in host cells. It is noteworthy that, when the PB2-627 site is changed from E to V, the virus can not only undergo contact transmission in ferrets but also undertake effective aerosol transmission, similar to that of the 17/H9N2^627K^ variant. In addition, PB2-627V can be maintained in ferrets post-infection during contact and aerosol transmission. The study on the H7N9 virus with a V/K/E polymorphism at position 627 also showed that V627 can transmit among ferrets (35). Thus, these features suggest that the dual adaptive PB2-627V in AIVs reduces the host barrier between birds and humans.

PB2-E627V has been identified in H5N1 viruses, where it was shown to increase viral replication in mammalian cells and virulence in mice (39). While in the background of a laboratory strain of H1N1 (PR8), it reduced viral replication in mammalian cells compared with PB2-627K (40). In H9N2 AIVs, PB2-627V variants appeared in the Middle East from 2000 to 2015 but were not stably maintained. The genotype of Middle East H9N2 viruses was different from the G57 genotype, which has been the dominant genotype of H9N2 virus circulation since 2010 (8, 41, 42). Under experimental conditions, PB2-627V in the Middle East H9N2 virus needed to operate in concert with PB2-E543D, PB2-A655V, and PB2-K526R to enhance viral replication in mammals (41), but these mutations were not identified in 17/H9N2, which we used in this study. Numerous adaptive mutations have recently been identified in the G57 H9N2 virus, including PB2-292, PB2-588, PA-356, M1-37, and HA-226 (15, 43, 44), in avian and human isolates (45–48). It is plausible that one or more of these adaptive changes in the G57 genotype exert epistatic effects to induce the emergence of the stable PB2-627V residue, but this remains to be tested.

Collectively, our results suggest that the recent variant PB2-E627V combines the functional characteristics of avian-like PB2-627E and human-like PB2-627K. It exhibits a crucial dual adaptation that can effectively infect and transmit in both avian and mammalian hosts, thereby overcoming the interspecific barrier between birds and humans. The presence of PB2-627V in multiple subtypes of AIVs undoubtedly has the potential to cause public health risk. We therefore propose that PB2-627V be included as a molecular marker to assess the zoonotic risk of AIVs.

## MATERIALS AND METHODS

### Sequence collection and alignment

The sequences of the PB2 gene were obtained from the GISAID database (http://www.gisaid.org). After removing the PB2 sequences of human H3N2 and H1N1pdm09 viruses and duplicate sequences, a total of 42,297 full-length PB2 sequences of IAVs published globally from 1994 to 2024 were obtained. Sequences were aligned using MUSCLE version 3.7 (49) via the CIPRES Science Gateway (50).

### Phylogenetic analyses

The phylogenetic tree of the H9N2 PB2 gene was constructed using a total of 4,523 PB2 sequences from H9N2 subtype IAVs. The phylogenetic tree of PB2-627V was constructed using a total of 394 PB2 sequences carrying the PB2-627V. IQ-TREE version 1.6 was used to construct the maximum likelihood phylogenetic trees, applying the best-fit general time-reversible model of nucleotide substitution with gamma-distributed rate variation among sites (GTR+I+G) and performing ultrafast bootstrap resampling analysis (1,000 replications). Phylogenetic trees were visualized and annotated using FigTree version 1.4.4, Adobe Illustrator 2021, and Interactive Tree of Life (iTOL) (51).

### Host transmission dynamics analyses

A maximum clade credibility (MCC) tree was performed on the PB2-627V cluster sequences (Fig. S1A) with BEAST version 1.10.4. Using the coalescent Bayesian Skygrid model, the GTR model of evolution and the strict clock were employed. The host jump patterns were interfered with by mapping the host types onto the time-scaled tree using an asymmetric discrete trait model (52). A Markov Monte Carlo chain is run for 30 million, sampled every 3,000, and the first 10% of the tree was removed as burn-in. Stationarity and mixing were checked with Tracer version 1.7.2. The MCC tree was finally generated by TreeAnnotator and visualized using FigTree version 1.4.4.

### PB2 protein structure simulation

Using the published structure of the H7N9 asymmetric polymerase dimer-ANP32A complex (PDB:8RMR) (28), we employed PyMOL to model the PB2-E627V and PB2-E627K mutations and analyze the resulting surface charge distribution of the protein.

### Cell lines

Human lung epithelial cell (A549) cells, kidney epithelial (293T) cells, chicken fibroblast (DF-1) cells, and Madin-Darby Canine Kidney (MDCK) cells were maintained in our laboratory. Chick embryo fibroblast cells were obtained from SPF embryonated chicken eggs as previously described (53). 293T cell line with a double knockout of ANP32A and ANP32B (DKO cell) was kindly provided by Xiaojun Wang from Harbin Veterinary Research Institute. Cells were cultured with Dulbecco’s modified Eagle medium (Gibco, New York, USA) supplemented with 10% fetal bovine serum (Gibco, Sydney, Australia) and 1% penicillin-streptomycin solution (MACGENE, Beijing, China).

### Influenza viruses

H9N2 AIV A/chicken/Hebei/HB17/2017 (17/H9N2) was characterized and maintained in our laboratory. Influenza H7N9 virus A/chicken/China/0606-12/2017 (17/H7N9) and influenza H3N8 virus A/chicken/Shandong/FE12/2022(H3N8) (22/H3N8) were isolated by our laboratory. Viruses were propagated in 9–11-day-old SPF embryonated chicken eggs at 35°C for 72 hours. Virus titers were determined by 50% tissue culture infectious dose (TCID_50_) on MDCK cells or 50% egg infectious dose (EID_50_) on SPF chicken eggs.

### Animals

Female SPF BALB/c mice aged 5–6 weeks and female SPF chickens aged 6–8 weeks were purchased from Vital River Laboratory Animal Technology Co., Ltd (Beijing, China). Mice were kept in individually ventilated cages, and chickens were kept in high-efficiency particulate air (HEPA)-filter isolators in the Animal Care Facilities at CAU. Male Angora ferrets (*Mustela putorius furo*), aged 5–6 months, serologically negative for currently circulating influenza viruses (H1, H3, H5, H7, and H9), and weighing >1.0 kg (ranging from 1.10 to 1.80 kg), were purchased from Angora LTD (Jiangsu, China). All ferrets were housed in wire cages placed inside HEPA-filter isolators. All animals were allowed free access to water and a standard chow diet and provided with a 12-hour light and dark cycle (temperature: 20°C–25°C, humidity: 40%–70%). All animals used were not involved in any other experimental procedure.

### Generation of viruses by reverse genetics

As described previously (18), recombinant viruses were generated using a plasmid-based reverse genetics system in the genetic background of 17/H9N2, 17/H7N9, and 22/H3N8. Mutations were introduced into the plasmids using PCR-based site-directed mutagenesis. To check for the absence of unintended mutations, all propagated viruses were subjected to complete sequencing.

### Polymerase activity assay

To compare the polymerase activities of different viral RNP complexes, a dual-luciferase reporter assay system (Promega, Madison, WI, USA) was employed (25). The PB2, PB1, PA, and NP gene segments of respective viruses were individually cloned into the pCDNA3.1 expression plasmid. One hundred twenty-five nanograms of each of the PB2, PB1, PA, and NP plasmids, as well as 10 ng of the pLuci luciferase reporter plasmid and 2.5 ng of the Raniera internal control plasmid, was transfected into 293T cells or DF-1 cells. The cell cultures were then incubated at 33°C or 37°C for 24 hours. The transfected cells were then lysed and analyzed for firefly and *Renilla* luciferase activities using a GloMax 96 microplate luminometer (Promega). The function of ANP32 was examined using a polymerase assay by co-transfection of DKO cells with different ANP32 proteins or empty vector (20 ng) at 33°C or 37°C for 24 hours. All the experiments were performed independently at least three times. The expression levels of polymerase proteins on different cell lines were detected by Western blotting, using influenza A PB2 antibody (Thermo, PA5-32221), Influenza A NP antibody (Abcam, ab128193), and anti-flag tag antibody (Sigma, F1804)

### Growth kinetics of viruses in A549 and CEF cells

A549 and CEF cells were cultured in 6-well plates and infected in triplicate with indicated viruses at a multiplicity of infection (MOI) of 0.1 (A549) or 0.01 (CEF). After a 2-hour incubation, cells were washed three times with DPBS and incubated at 37°C in 5% CO_2_. Supernatants were collected at 12, 24, 36, 48, and 60 hours post-infection, and virus titers were determined by TCID_50_ assays on MDCK cells.

### Quantitative real-time PCR assay

The levels of mRNA and viral RNA (vRNA) were determined in A549 cells and DF-1 cells infected in triplicate with viruses (17/H9N2^627E^, 17/H9N2^627V^, or 17/H9N2^627K^) at an MOI of 0.1 (A549 cells) or an MOI of 0.001 (DF-1 cells). Total RNA was extracted from infected cells by using TRIzol reagent according to the manufacturer’s instructions (Invitrogen). For the detection of mRNA and vRNA, the oligo(dT) primer and primer uni-12 (5′-AGCAAAAGCAGG-3′) were used, respectively, to generate cDNAs from 1 µg of total RNA per sample by using Superscript III First-Strand Synthesis SuperMix (Invitrogen). The quantitative real-time PCR (qRT-PCR) mixture for each reaction sample consisted of 10 µL of 2× SYBR green PCR master mix (Applied Biosystems), 7 µL of nuclease-free water, 0.5 µL of each primer, and 2 µL of the cDNA template. qPCR was conducted using a 7500 real-time PCR system (Applied Biosystems) with the following program: 1 cycle at 95°C for 10 min, followed by 40 cycles at 95°C for 15 s and 60°C for 1 min. Expression values for each gene, relative to *β-actin*, were calculated by using the 2^-ΔΔCT^ method. Each qPCR sample contained three technical replicates. Primers for the amplification of the *β-actin* and PB2 genes are as follows: forward primer: 5′-AGAGCTACGAGCTGCCTGAC-3′ and reverse primer: 5′-CGTGGATGCCACAGGACT-3′ for *β-actin*; forward primer: 5′- GGAACAGGAATGGACCGACA-3′ and reverse primer 5′-ACTGAGATCTGCATGACCCG-3′ for PB2.

### Mouse challenge studies

Groups of eight mice were anesthetized by intramuscular injection of Zoletil 50 (Zoletil; Virbac SA, Carros, France) at a dose of 20 mg/kg of body weight and inoculated intranasally (i.n.) with 10^6^ TCID_50_ of test virus or PBS in a total volume of 50 µL. At 3 days post-infection, three mice from each group were euthanized, and lung and nasal turbinate tissues were collected for virus titration in MDCK cells by TCID_50_ assay. Lungs of mice infected with the indicated viruses at 3 dpi were collected for histopathology and immunohistochemistry (IHC). The remaining five animals were monitored in the remaining 2 weeks for disease progression, weight change, and mortality. Mice that experienced a weight loss exceeding 25% of their original weight were humanely euthanized.

### Chicken challenge studies

Experimental procedures for virus infection of chickens were under a previously described protocol (54). Groups of six chickens were inoculated i.n. with 10^6^ EID_50_ of the indicated virus or PBS in a volume of 200 µL. At 3 dpi, three chickens from each group were euthanized, and lung and nasal turbinate tissues were collected for virus titration by EID_50_ assay. Lungs of chickens at 3 dpi were collected for histopathology and IHC. The remaining three chickens were monitored, and tracheal swabs were taken at 2, 4, 6, and 8 dpi for virus titration by EID_50_ assay.

### Histopathology and IHC analysis

Lungs were fixed in 10% buffered formalin, embedded in paraffin, sectioned, and stained with hematoxylin and eosin. Tissue sections were also immunostained with a viral NP monoclonal primary antibody (ab20343, Abcam, Beijing, China). The secondary antibody used was conjugated to horseradish peroxidase (HRP), and the color reaction was based on the use of an HRP reaction kit (ZSGB-BIO, Beijing, China). Histopathological examination was conducted in a blinded manner by two experienced experimental pathologists. We utilized QuPath to analyze three randomly selected 1,000 × 1,000 pixel fields of view in the IHC-scanned images, quantifying positive cells and determining the percentage of IHC-positive cells.

### Virus passage in chickens

For virus passage in chickens, as depicted in Fig. 4A, groups of two SPF chickens were inoculated at passage 1 (P1) with 10^6^ EID_50_ of the indicated virus in a volume of 200 µL. At three dpi, two naive chickens were co-housed with the P1 chickens to establish the P2 generation. At 6 dpi of P1 chickens, lung tissues of P1 chickens were collected and homogenized in 500 µL of cold PBS, and another two naive chickens were introduced as P3. The same sequential steps were used for passage up to the P5.

### Ferret transmission experiments

The transmission study consisted of groups of three ferrets, following a previously described protocol (34, 55) comprising one infected, one direct contact, and one respiratory droplet contact group. Ferrets were housed in wire cages inside isolators, with each isolator approximately 15 cubic feet in size. Each ferret was sedated with ketamine (20 mg/kg of body weight) and xylazine (1 mg/kg of body weight) via intramuscular injection and inoculated intranasally with 10^6^ TCID_50_ of 17/H9N2^627E^, 17/H9N2^627V^, or 17/H9N2^627K^ virus in a volume of 500 µL, delivered at 250 µL per nostril. Twenty-four hours later, two naive ferrets were introduced into the isolator, with the direct contact ferret placed in the same cage as the infected ferret, while the respiratory droplet contact ferret was placed in a cage separated by a wire mesh wall to prevent physical contact.

Individual weight was monitored throughout the study. Nasal washes were collected from the ferrets by 2 mL virus transport medium every other day for 14 days and titrated for viruses in MDCK cells by TCID_50_ assays. Additionally, 200 µL nasal washes were used for RNA extraction and deep sequencing. At 21 dpi, blood samples of ferrets were collected to determine seroconversion using the HI Assay.

### Next-generation sequencing

A total of 54 ferret nasal wash samples, 6 chicken lung samples, and 3 viruses (17/H9N2^627E^, 17/H9N2^627V^, and 17/H9N2^627K^) were subjected to whole-genome sequencing. Viral RNA was harvested from the samples by using the QIAamp Viral RNA Mini Kit (Qiagen). Multiplex reverse transcription-PCR amplification of all eight influenza virus genome segments was performed on RNA samples using PrimeScript One Step RT-PCR Kit (Takara RR055A) and primers Uni12/Inf1 (5′-ACGCGTGATCAGCAAAAGCAGG-3′), Uni12/Inf3 (5′-ACGCGTGATCAGCGAAAGCAGG-3′), and Uni13/Inf1 (5′-ACGCGTGATCAGTAGAAACAAGG-3′). As previously described (34), PCR products were purified on spin columns and quantified by using the Quant-It Pico Green kit (Invitrogen, Carlsbad, USA). The purified amplicons were pooled in equimolar concentrations and paired-end sequenced (23,150) on an Illumina NovaSeq Platform according to standard protocols. Sequencing was performed using 200 or 250 bp paired-end reads, and the sequencing depth for avian influenza virus isolates was about 0.2 G per sample. NGS findings were validated by qRT-PCR. Raw NGS reads were processed by filtering out low-quality reads (eight bases with quality < 66), adapter-contaminated reads (with >15 bp matched to the adapter sequence), poly-Ns (with eight Ns), and duplication and host-contaminated reads (SOAP2 [version 2.21]; less than five mismatches). Sanger sequences of parental viruses were chosen as reference sequences. Burrows-Wheeler Alignment Tool (version 0.7.12) and SAMtools (version 1.4) were then used to perform reference-based assembly. Data analyses were performed using a custom quality-based variant detection pipeline. Due to potential contamination identified in sequencing samples from the second and third parallel experiments during subsequent analysis, all data from these experiments were excluded from the final results. Only the deep sequencing results of the three viruses from the first experiment in ferrets (#1, Table S4) are presented.

To detect the genomic variants, the iSNVs were identified as following standards: (i) Bonferroni-corrected *P* value < 0.01; (ii) average MapQ score on variant reads > 30; (iii) average phred score on variant positions > 35; (iv) average position of variant call on a read > 15 and <135; (v) reads number > 3,000; and (vi) variant frequency >1%. The iSNV frequency is the proportion of iSNVs in the whole genome. Based on the reported algorithm, the nucleotide diversity π was calculated in each set of samples (56, 57).

### Quantification and statistical analysis

Graphing and statistical analyses were performed using GraphPad Prism 8 (GraphPad Software, San Diego, CA, USA; https://www.graphpad.com/). Experimental groups were statistically compared by analysis of variance. *P* value < 0.05 was considered to indicate statistical significance.

## Data Availability

All genetic variation data generated in this study are available in the Zenodo repository. This includes data from chicken passage studies (doi: 10.5281/zenodo.12730083) and ferret transmission experiments (doi: 10.5281/zenodo.15745176). All other data supporting the findings of this study are available in the main text or the supplemental material.

## SUPPLEMENTAL MATERIAL

Supplemental material jvi.00853-25-s0001.docx Figures S1 to S8; Tables S1 to S4.

ASM does not own the copyrights to Supplemental Material that may be linked to, or accessed through, an article. The authors have granted ASM a non-exclusive, world-wide license to publish the Supplemental Material files. Please contact the corresponding author directly for reuse.
